# Electride support boosts nitrogen dissociation over ruthenium catalyst and shifts the bottleneck in ammonia synthesis

**DOI:** 10.1038/ncomms7731

**Published:** 2015-03-30

**Authors:** Masaaki Kitano, Shinji Kanbara, Yasunori Inoue, Navaratnarajah Kuganathan, Peter V. Sushko, Toshiharu Yokoyama, Michikazu Hara, Hideo Hosono

**Affiliations:** 1 Materials Research Center for Element Strategy, Tokyo Institute of Technology, 4259 Nagatsuta, Midori-ku, Yokohama 226-8503, Japan; 2 Materials and Structures Laboratory, Tokyo Institute of Technology, 4259 Nagatsuta, Midori-ku, Yokohama 226-8503, Japan; 3 Department of Physics and Astronomy, University College London, Gower Street, London WC1E 6BT, UK; 4 Fundamental and Computational Sciences Directorate, Pacific Northwest National Laboratory, Richland, Washington 99352, USA; 5 ACCEL, Japan Science and Technology Agency, 4-1-8 Honcho, Kawaguchi, Saitama 332-0012, Japan; 6 Frontier Research Center, Tokyo Institute of Technology, 4259 Nagatsuta, Midori-ku, Yokohama 226-8503, Japan

## Abstract

Novel approaches to efficient ammonia synthesis at an ambient pressure are actively sought out so as to reduce the cost of ammonia production and to allow for compact production facilities. It is accepted that the key is the development of a high-performance catalyst that significantly enhances dissociation of the nitrogen–nitrogen triple bond, which is generally considered a rate-determining step. Here we examine kinetics of nitrogen and hydrogen isotope exchange and hydrogen adsorption/desorption reactions for a recently discovered efficient catalyst for ammonia synthesis—ruthenium-loaded 12CaO·7Al_2_O_3_ electride (Ru/C12A7:e^−^)—and find that the rate controlling step of ammonia synthesis over Ru/C12A7:e^−^ is not dissociation of the nitrogen–nitrogen triple bond but the subsequent formation of N–H_*n*_ species. A mechanism of ammonia synthesis involving reversible storage and release of hydrogen atoms on the Ru/C12A7:e^−^ surface is proposed on the basis of observed hydrogen absorption/desorption kinetics.

## Abstract (web summary)

Development of catalysts that enhance dissociation of the nitrogen–nitrogen triple bond will reduce costs of ammonia production. Here, the authors study ammonia synthesis over a ruthenium loaded electride catalyst and show that the rate-determining step is shifted to nitrogen–hydrogen bond formation.

## Graphical abstract

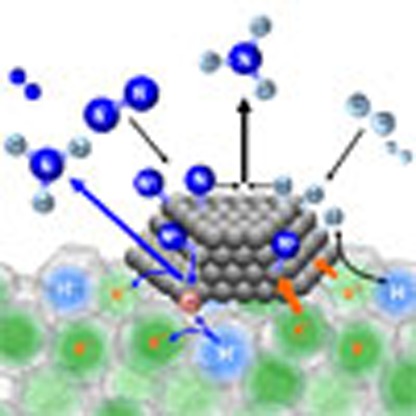

Over the past century, industrial ammonia synthesis has been carried out by the Haber–Bosch process that uses iron-based catalysts and requires high temperatures (673–873 K) and pressures (20–40 MPa; ref. 1). The primary difficulty in ammonia synthesis originates from the character of the N≡N bond, which is the strongest bond among diatomic molecules. The role of the catalyst is to lower the dissociation energy of the N≡N bond. Both Fe and ruthenium (Ru) are well-known catalysts for ammonia synthesis, and their catalytic activity is significantly enhanced by basic promoters such as alkali and alkaline earth metal oxides (Cs_2+*x*_O, K_2_O, BaO_*x*_ and so on)^2^,^3^,^4^,^5^,^6^. Such enhancement of the catalytic activity is explained by the electron transfer from the promoter to the antibonding π-orbitals of N_2_ through the metal catalyst and is referred to as electronic promoting effect^2^,^7^. However, the promoting effect alone is not sufficient to facilitate ammonia synthesis under mild conditions because the chemical composition of these promoters—hydroxide or oxides^8^,^9^—constrains their ability to donate electrons. Although pure alkali and alkaline earth metals drastically enhance the catalytic activity of Fe and Ru^10^, they are chemically unstable under ammonia synthesis conditions. Several attempts were undertaken in the past decades to clarify the reaction mechanism of ammonia synthesis over the Fe and Ru catalysts. The following three possibilities for the rate-determining step (RDS) of ammonia synthesis are suggested: (1) the dissociative adsorption of N_2_ [N_2_→2N(ad)] (ref. 11); (2) the surface reaction of adsorbed species [N(ad)+H(ad) →NH(ad)]^12^ and (3) the desorption of adsorbed ammonia [NH_3_(ad)→NH_3_(g)]^13^. At present, it is widely recognized that N_2_ dissociation is the RDS of ammonia synthesis, irrespective of the catalyst.

It was recently reported that 12CaO·7Al_2_O_3_ electride (C12A7:e^−^), the first room temperature stable electride, functions as an efficient electronic promoter for Ru catalyst^14^. C12A7:e^−^ has a unique crystal structure consisting of a positively charged framework having the chemical formula [Ca_24_Al_28_O_64_]^4+^, and four extra-framework electrons, accommodated in the cages as the counter ions^15^. It has been demonstrated that the extra-framework electrons can be replaced by various anions, such as OH^−^ (refs 16, 17), O^−^ (ref. 18), F^−^ (ref. 17), Cl^−^ (refs 17, 19) and H^−^ (ref. 20). As reported earlier^14^, Ru/C12A7:e^−^ exhibits an order of magnitude higher catalytic activity, as defined by the turnover frequency (TOF), for ammonia synthesis than Ru/C12A7:O^2−^ and the conventional Ru catalysts. C12A7:e^−^ has a low work function (2.4 eV) comparable to that of potassium metal; nevertheless, this material is chemically and thermally stable^21^. Electrons transferred from C12A7:e^−^ to the supported Ru nanoparticles shift the Fermi level up with respect to that in isolated Ru, which in turn allows for the effective electron donation from Ru/C12A7:e^−^ to the antibonding π-orbitals of N_2_. These electrons are replenished via the Ru contact with the C12A7:e^−^ substrate, thus providing persistent catalytic activity. In addition, the ability of C12A7 to store and release hydrogen reversibly^22^ prevents the poisoning of the Ru surface by the hydrogen adatoms. Furthermore, the Ru/C12A7:e^−^ catalyst is markedly different from ammonia synthesis catalysts that have been extensively studied so far in that it exhibits ca. 0.5 reaction order for N_2_, while the reaction orders for conventional catalysts, including Fe- and Ru-based materials, are 0.8–1.0. This observation suggests that N adatoms populate the Ru/C12A7:e^−^ surface more densely than surfaces of other catalysts. While the origin of this effect and its specificity to the electride-supported Ru nanoparticles have not been established yet, it is clear that the details of the reaction mechanism on Ru/C12A7:e^−^ are different from those on other catalysts.

Herein, we demonstrate, by using N_2_ isotopic exchange reaction, hydrogen adsorption/desorption reaction and density functional theory (DFT) calculations that N_2_ cleavage is not the RDS in ammonia synthesis. Fast N_2_ cleavage is ensured by highly efficient electron transfer from C12A7:e^−^ to N_2_ molecules adsorbed on the Ru nanoparticles. As a result, the bottleneck in the NH_3_ synthesis reaction is shifted from the N≡N bond dissociation to the formation of N–H_*n*_ species.

## Results

### N_2_ cleavage on Ru/C12A7:e^−^

The details of dissociative adsorption and associative desorption of N_2_ molecules on the Ru/C12A7:e^−^ catalyst were examined through an N_2_ isotopic exchange reaction (equation 1)


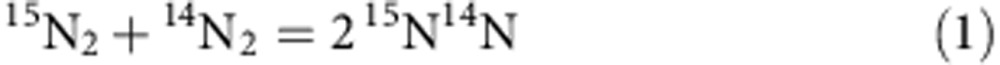


under the conditions similar to those used in ammonia synthesis (613–673 K) at 0.1 MPa. During this reaction, both dissociative adsorption and recombinative desorption of N_2_ proceed on the catalyst surface and the activation energy of the former is smaller than that of the latter^23^,^24^,^25^. It is well known that efficient catalysts for ammonia synthesis also exhibit high catalytic performance for the N_2_ isotopic exchange reaction^10^,^26^,^27^,^28^. Supplementary Figure 1a shows the Arrhenius plots for N_2_ isotopic exchange reaction rate (*R*) of the tested Ru catalysts. The Ru particle sizes, dispersion, activities and TOFs are summarized in Table 1. Ru/C12A7:e^−^ exhibits a remarkable activity for the reaction among tested catalysts despite its low surface area (Table 1). This is consistent with the results for NH_3_ synthesis as shown in Supplementary Table 1. While Ru-Cs/MgO has been the most active catalyst for ammonia synthesis among conventional Ru-based materials, Ru/C12A7:e^−^ surpasses Ru-Cs/MgO not only in catalytic activity per catalyst weight but also in TOF: the turnover frequency of Ru/C12A7:e^−^ exceeds that of Ru-Cs/MgO at 633 K by the factor of 60. In addition, the activation energy in the case of the electride catalyst is less than half of the corresponding values found for other materials. We conclude that a faster isotopic N_2_ exchange reaction with a smaller energy barrier proceeds on Ru/C12A7:e^−^, and the origin of this effect can be attributed to the ability of C12A7:e^−^ to donate electrons at the concentrations and rates exceeding those of alkali-based electron promoters. For comparison, Ru/CA (CaO·Al_2_O_3_) has a moderate catalytic activity in the tested catalysts. The crystal structure of CA is composed of a fully polymerized network of corner-shared AlO_4_ tetrahedra arranged into a tridymite lattice, with Ca^2+^ occupying large voids within this network^29^. It can be viewed as a negatively charged framework, lacking intrinsic nanostructure, stuffed with positively charged ions. Catalytic performance of such an oxide is inferior even to that of C12A7:O^2−^.

**Figure 1:**
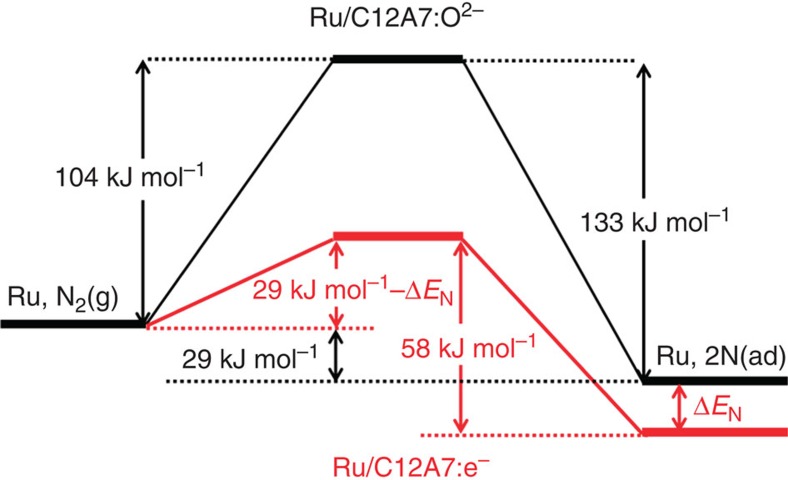
Energy profile of N_2_ dissociation. Potential energy profile for dissociative adsorption of N_2_ and associative desorption of N_2_ on Ru/C12A7:e^−^ and Ru/C12A7:O^2−^. These values were estimated from the results of N_2_ exchange and ammonia synthesis reactions. N_2_(g) and N(ad) represent N_2_ in gas phase and adsorbed nitrogen atom, respectively.

The Arrhenius plots for ammonia synthesis rate (*r*) over the tested catalysts are shown in Supplementary Fig. 1b. Apparent activation energy estimated from this plot corresponds to the activation energy in the RDS in the overall chemical process. In ammonia synthesis, this step has been considered to be N_2_ cleavage^30^. Again, Ru/C12A7:e^−^ has the smallest apparent activation energy among the tested catalysts, and its value (49 kJ mol^−1^) is 40–60% of that found for other catalysts, which is consistent with highly efficient ammonia formation over Ru/C12A7:e^−^ (ref. 14). Table 2 summarizes the relevant thermodynamic data, including activation energies for N_2_ desorption estimated by temperature-programmed desorption (TPD) and difference (Δ*E*) in the apparent activation energy between N_2_ isotopic exchange reaction and ammonia formation. In Ru/C12A7:e^−^ and Ru-Cs/MgO, there is no significant difference between activation energies estimated from N_2_ isotopic exchange reaction and N_2_ desorption^27^,^31^, indicating that the RDS of N_2_ isotopic exchange reaction is the recombination of N adatoms. The data in Table 2 also show that Δ*E* of Ru/C12A7:e^−^ (9 kJ mol^−1^) is significantly smaller than that for Ru/C12A7:O^2−^ (29 kJ mol^−1^) and other catalysts (36–78 kJ mol^−1^). In particular, in the case of Ru/C12A7:O^2−^, the energy of N adatoms on the Ru is lower than that of gas phase N_2_ by 29 kJ mol^−1^, as shown in Fig. 1. Since adsorbed N atoms have a positive electron affinity, electron transfer from C12A7:e^−^ substrate to Ru and then from Ru to N adatoms stabilizes them, thus, resulting in a larger adsorption energy of N adatoms on Ru/C12A7:e^–^ than on Ru/C12A7:O^2–^ (ref. 32). As a result, the actual activation energy for N_2_ cleavage on Ru/C12A7:e^−^ is <29 kJ mol^−1^ and the value is reduced by the difference (Δ*E*_N_) between the binding energies of N adatoms to Ru/C12A7:e^–^ and to Ru/C12A7:O^2–^. This small energy barrier for N_2_ dissociation strikingly differs from the apparent activation energy for ammonia synthesis over Ru/C12A7:e^−^ (49 kJ mol^−1^).

**Figure 2:**
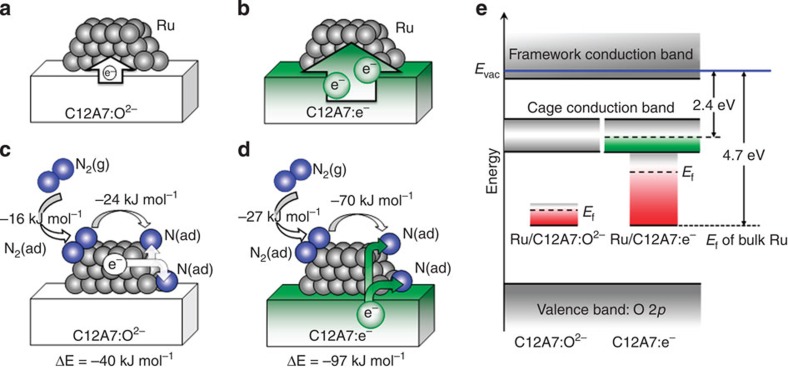
***Ab initio*** **simulations of N**_**2**_ **interaction with the Ru/C12A7 catalysts.** Character of the charge redistribution between C12A7 substrate and deposited Ru clusters for the stoichiometric (**a**) and electride (**b**) C12A7. (**c**,**d**) Adsorption energies of N_2_ on C12A7-supported Ru, charge transfer in the process of N_2_ dissociation (N_2_(g)+Ru→2N(ad)+Ru) and the corresponding energy gain (Δ*E*). In Ru/C12A7:O^2–^ system (**c**), N_2_ and N accept electron charge from the Ru cluster, making it positively charged. In Ru/C12A7:e^–^ (**d**), the electron charge is transferred from the substrate, leaving the Ru cluster nearly neutral. N_2_(g), N_2_(ad) and N(ad) represent N_2_ in gas phase, adsorbed N_2_, and adsorbed nitrogen atom, respectively. (**e**) Electronic structure: the Fermi level (*E*_f_) of Ru on C12A7:O^2–^ is similar to that of bulk Ru (4.7 eV) and that of the Ru/C12A7:e^–^ is determined by the charge transfer from the cage conduction-band electrons of C12A7:e^–^ (2.4 eV). *E*_vac_ denotes vacuum level.

Electron transfer from C12A7:e^−^ to N_2_ molecules and their adsorption energies were also examined by *ab initio* (DFT) simulations using a model system (Fig. 2, and Supplementary Figs 2 and 3). It can be seen that the charge (*Q*) transfer from C12A7:e^−^ surface to a Ru cluster (*Q*(Ru_4_)=−1.66e), a model active site corresponding to an edge of a Ru-nanoparticle, is distinctly larger than that in the case of Ru loaded on C12A7:O^2−^ surface (*Q*(Ru_4_)=−0.43e) (Supplementary Figs 2 and 3). Such electron transfer raises the Fermi level (*E*_f_) of Ru nanoparticles and the *E*_f_ of Ru on C12A7:e^−^ is located at a higher level, below the *E*_f_ of C12A7:e^−^, than that of Ru/C12A7:O^2−^ (Fig. 2e). When N_2_ molecules are adsorbed on Ru particles, the charges of the molecules on Ru/C12A7:O^2−^ and Ru/C12A7:e^−^ increase to −0.69 and −0.96e, respectively. The adsorption energy is larger for Ru/C12A7:e^−^ (27 kJ mol^−1^) than that for Ru/C12A7:O^2−^ (16 kJ mol^−1^). In addition, the resulting N adatoms are much more stabilized on Ru/C12A7:e^−^ than on Ru/C12A7:O^2−^. Dissociative adsorption of N_2_ molecule proceeds with the calculated energy gain of 40 kJ mol^−1^ for Ru/C12A7:O^2−^ and 97 kJ mol^−1^ for Ru/C12A7:e^−^, indicating that dissociative adsorption of N_2_ on Ru catalyst is remarkably enhanced by electron donation from C12A7:e^−^ (Fig. 2c,d). The results of these calculations substantiate the experimental observations, as shown in Supplementary Fig. 1a, and support the energy landscape displayed in Fig. 1.

Ammonia synthesis over heterogeneous catalysts includes the steps of N_2_ cleavage and formation of N–H_*n*_ species (NH, NH_2_ and NH_3_); the former step has been considered to require larger activation energy than the latter in all conventional catalysts reported so far^30^. It is well known that H_2_ dissociation barrier on Ru catalyst is negligibly small^33^. For this reason, it has been generally accepted that N_2_ cleavage is the rate-limiting step of ammonia synthesis from N_2_ and H_2_. The analysis presented here indicates that the RDS of ammonia synthesis on the electride catalyst is not dissociative N_2_ adsorption (the estimated barrier is <29 kJ mol^−1^) but one of the subsequent steps. Thus, we conclude that the RDS for the Ru/C12A7:e^−^ catalyst is in the formation of N–H_*n*_ species.

### Kinetic analysis

The energy profile for N_2_ dissociation reaction must also make distinction in kinetics between Ru/C12A7:e^−^ and conventional catalysts. The reaction orders for N_2_, H_2_ and NH_3_ in various ammonia synthesis catalysts^34^,^35^,^36^, including Ru/C12A7:e^−^, Ru/C12A7:O^2−^ and Ru/C12A7:H^−^, are summarized in Table 3. The rate of ammonia synthesis (*r*) is given by the following equation^34^:


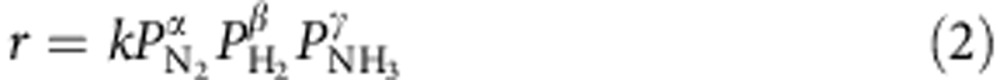


Here, *k* is the rate constant and *α*, *β* and *γ* represent the reaction orders for N_2_, H_2_ and NH_3_, respectively. While the reaction mechanism cannot be determined solely by reaction orders, they provide useful insight into the atomistic origin of the reaction mechanism. Variations in the rate of ammonia formation for Ru/C12A7:e^−^, Ru/C12A7:O^2−^, Ru/C12A7:H^−^ and Ru/CA with N_2_ and H_2_ pressures are shown in Supplementary Fig. 4.

The reaction order for N_2_ is 0.8–1.0 in all conventional heterogeneous catalysts (Table 3) as is already well known, but the reaction order in Ru/C12A7:e^−^ is only 0.46. A possible explanation for the reaction order is that Ru/C12A7:e^−^ is more densely populated with N adatoms than other catalysts. In conventional catalysts, where N_2_ cleavage is the rate-limiting step, surface N adatom concentration on a transition metal surface is limited by the rate of N_2_ cleavage, so that the reaction order for N_2_ is ~1. However, N adatom density on Ru/C12A7:e^−^ is not constrained by dissociative N_2_ adsorption and, therefore, can be higher than that on other catalysts, resulting in the reaction order of 0.46. In other words, the low reaction order is consistent with our conclusion that N_2_ cleavage is not the RDS of ammonia synthesis on Ru/C12A7:e^−^. The reaction orders for N_2_ on Ru/CA, Ru/C12A7:O^2−^ and Ru/C12A7:H^−^ are in the range of 0.82–1.0 (Table 3), indicating that although these materials belong to the same CaO–Al_2_O_3_ family and support the same conventional catalyst (Ru), the lack of significant electronic density of states at a shallow level makes them clearly distinct from Ru/C12A7:e^−^.

Second feature to be noted in Table 3 is that the reaction order for H_2_ of all Ru-based catalysts other than Ru/C12A7:e^−^ is between −1 and 0. The negative values are due to so-called hydrogen poisoning on Ru: dissociative adsorption of H_2_ is preferred over N_2_ cleavage on Ru, thus, suppressing efficient ammonia synthesis under high pressures^37^,^38^. From the view point of yield, storage and transportation, high pressure is obviously favourable for efficient ammonia production. However, ammonia synthesis commensurate with increase in pressure is not expected in conventional Ru-based catalysts because of severe hydrogen poisoning on Ru surfaces. It is a major reason why the Fe-based catalysts used in the Haber–Bosch process have not been replaced by the Ru catalysts for over a century. In contrast to the conventional catalysts, Ru/C12A7:e^−^ exhibits +1 reaction order with respect to H_2_, meaning that it is not subject to hydrogen poisoning, and, instead, it maintains high catalytic performance even under high pressure. In fact, ammonia formation activity of Ru/C12A7:e^−^ increases proportionally to the total pressure^14^. C12A7:e^−^ can incorporate H atoms into the cages as H^−^, which can be described by the reaction between H atoms and cage electrons: H^0^+e^−^→H^−^. The electrons remain in the cages when H atoms are released from the cages, hydrogen release reaction H^−^→H^0^+e^−^ (ref. 22). This reversible hydrogen storage–release reaction in Ru/C12A7:e^−^ drastically reduces hydrogen poisoning of the catalyst and controls the rate of ammonia synthesis. However, such a reaction does not proceed in Ru/C12A7:H^−^ because no extra-framework electrons are available to stabilize H^–^. Thus, it is reasonable that H_2_ reaction order for Ru/C12A7:H^−^ is −0.63. Supplementary Fig. 5 also shows that Ru/C12A7:H^−^, where all cage electrons are replaced by H^−^ anions, is much inferior to Ru/C12A7:e^−^ in catalytic activity. This result indicates that H^−^ ions encaged in C12A7 have no promoting effect on the catalytic activity. For Ru/C12A7:e^−^, extra-framework H^−^ ions formed in close proximity to the Ru catalyst by hydrogen spillover, and H atoms from cage H^−^ anions readily react with N adatoms on Ru, resulting in ammonia formation and cage electrons. Therefore, electrons in Ru/C12A7:e^−^ are not fully substituted with H^−^ ions during ammonia synthesis. In fact, no decrease in activity of Ru/C12A7:e^−^ was observed even after 75 h of ammonia synthesis^14^. As for the reaction order of NH_3_, Ru/C12A7:e^−^ showed a large negative value (−1.0). Such a negative value indicates that Ru/C12A7:e^−^ exhibits high catalytic performance for ammonia decomposition^31^, thus, effectively, inhibiting the formation of NH_3_. While Ru catalysts are typically less inhibited by NH_3_ than Fe and Mo catalysts^38^, the produced ammonia needs to be removed from the catalyst bed because its catalytic activity is reduced at high N_2_ and H_2_ conversions.

**Figure 3:**
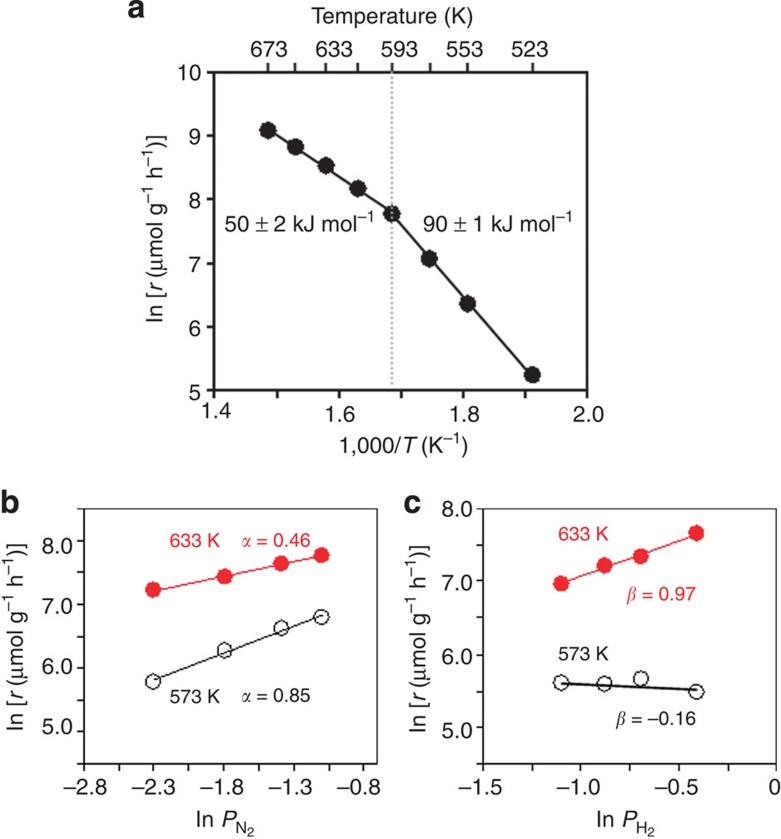
Kinetic analysis of ammonia synthesis over Ru/C12A7:e^−^ catalysts. (**a**) Temperature dependence of the rate of ammonia synthesis over Ru/C12A7:e^−^ catalysts at an atmospheric pressure (catalyst=0.025 g, H_2_:N_2_=3:1, flow rate=60 ml min^−1^) (**b**,**c**) Dependence of NH_3_ synthesis rate on the partial pressures of (**b**) N_2_ and (**c**) H_2_ at 573 (open circles) and 633 K (filled circles) under atmospheric pressure. *α* And *β* represent the reaction orders for N_2_ and H_2_ in equation 2, respectively.

**Figure 4:**
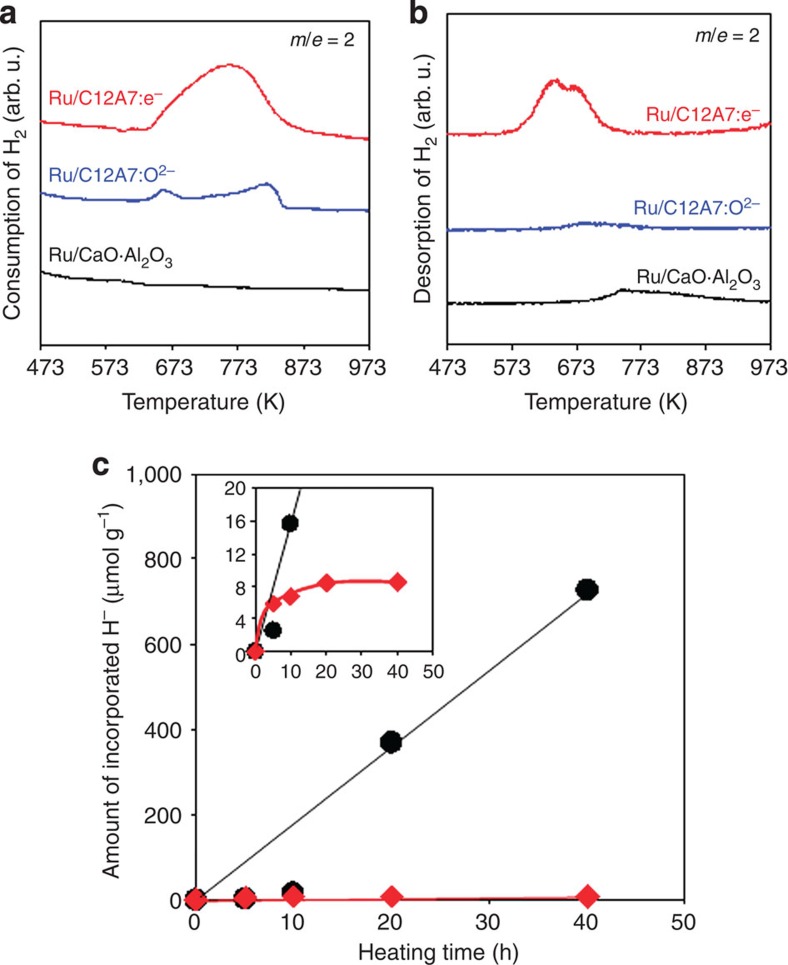
Hydrogen incorporation into C12A7:e^−^. (**a**) H_2_ TPA profiles of Ru/C12A7:e^−^, Ru/C12A7:O^2−^ and Ru/CA. The TPA experiment was performed with a dilute mixture of H_2_ (5%) in Ar using a total flow of 10 ml min^−1^. (**b**) H_2_ TPD profiles of Ru/C12A7:e^−^, Ru/C12A7:O^2−^ and Ru/CA. The TPD experiment was performed with Ar using a total flow of 10 ml min^−1^. (**c**) Amount of incorporated H^−^ ions in Ru/C12A7:e^−^ after heat treatment in H_2_ atmosphere; black circle: Ru/C12A7:e^−^ heated in H_2_ (75 kPa) and Ar (25 kPa) gas flow at 633 K. Red diamond: Ru/C12A7:e^−^ heated in H_2_ (75 kPa) and N_2_ (25 kPa) gas flow at 633 K. Inset shows the enlarged profiles. Neutral hydrogen species such as H^0^ and H_2_ are metastable C12A7 because the cage wall is positively charged^22^.

In order to elucidate the reaction mechanism of ammonia synthesis over Ru/C12A7:e^−^, deuterated ammonia (ND_3_) formation from N_2_ and D_2_ was examined. Supplementary Fig. 6 shows the Arrhenius plots for ammonia (NH_3_ or ND_3_) formation rate over Ru-Cs/MgO and Ru/C12A7:e^−^. The rates of ND_3_ formation are higher than those of NH_3_ in both catalysts, that is, *k*(D)/*k*(H)>1 (where *k*(D) and *k*(H) are rate constants of the formation of ND_3_ and NH_3_, respectively). This is due to the inverse isotope effect that has been frequently observed for ammonia formation on Fe and Mo metal catalysts^39^,^40^. To rationalize this result, we note that the dissociation of N_2_ is suppressed by adsorbed species such as N(ad) and NH(ad), and that the deuterium system gives a lower concentration of these suppressing species on the catalyst surface than the hydrogen system. There is no large difference in apparent activation energy between ND_3_ and NH_3_ formation by both catalysts, indicating that the difference between ammonia synthesis using H_2_ and D_2_ is mainly determined by the pre-exponential factor of the rate. Therefore, the reaction mechanism is not affected by deuterated ammonia synthesis. It should be noted that the Arrhenius plot for ammonia formation by Ru/C12A7:e^−^ has an inflection point at 593 K (Fig. 3a). The activation energy of Ru/C12A7:e^−^ below 593 K was estimated to be 91 kJ mol^−1^, which is comparable to that of conventional Ru catalysts, while it is ~50 kJ mol^−1^ at temperatures above 593 K. This finding indicates that the dominant reaction pathway on Ru/C12A7:e^−^ is switched by the reaction temperature. As shown in Fig. 3b,c, the reaction orders for N_2_ and H_2_ on Ru/C12A7:e^−^ at 573 K are 0.85 and −0.16, respectively, and there is no significant difference in activation energy and reaction order at temperatures below 593 K between conventional Ru catalysts and Ru/C12A7:e^−^: Ru/C12A7:e^−^ is also subject to hydrogen poisoning below this temperature, as well as other Ru catalysts. As shown in temperature-programmed absorption (TPA) and TPD experiments (Fig. 4), both hydrogen storage and release reactions proceed on Ru/C12A7:e^−^ at temperatures above 593 K. These results strongly suggest that the change in the reaction mechanism over Ru/C12A7:e^−^ is significantly influenced by the reversible hydrogen storage–release properties of Ru/C12A7:e^−^ (the details are discussed in the next section).

### Hydrogen storage–release reaction over Ru/C12A7:e^−^

**Figure 5:**
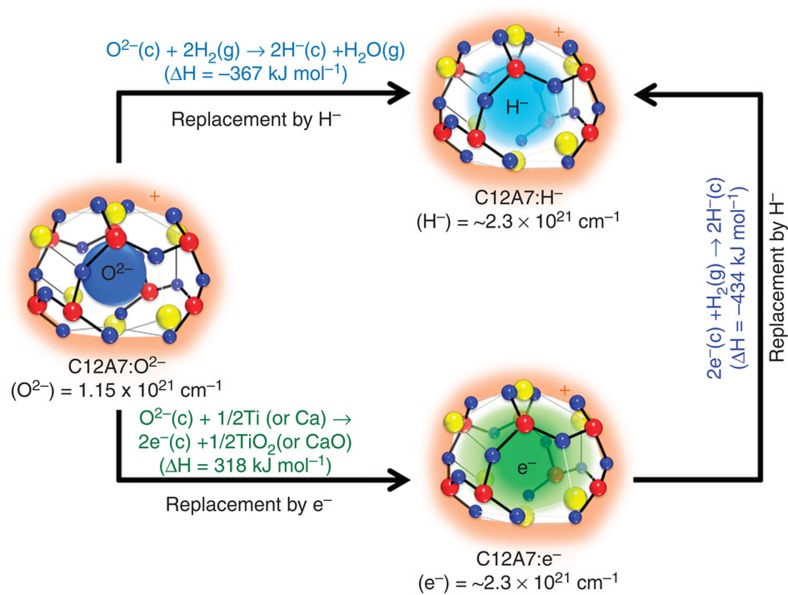
Enthalpy changes for extra-framework species in 12CaO·7Al_2_O_3_ (C12A7). C12A7 has two chemical formula units/cubic unit cell. The extra-framework O^2−^ ions are loosely bound to the positively charged framework [Ca_24_Al_28_O_64_]^4+^ to keep electroneutrality. The O^2−^ ions can be partially or completely replaced by e^−^ and H^−^ ions. The enthalpies (Δ*H*) for e^−^ or H^−^ ions formation in the cage of C12A7:O^2−^ are 318 and −367 kJ mol^−1^, respectively. C12A7:e^−^ easily reacts with hydrogen gas to form H^−^ ions in the cage (Δ*H*=−434 kJ mol^−1^) as compared with C12A7:O^2−^. ‘c’ and ‘g’ denote the species in a cage and gas phase, respectively.

C12A7 has unique anion-exchange ability, for example, extra-framework O^2−^ ions can be replaced by e^−^ and/or H^−^ ions (Fig. 5). The replacement of the extra-framework O^2−^ ions with electrons can be achieved by reacting C12A7:O^2−^ with Ti or Ca metals under vacuum at high temperatures, which results in the formation of surface TiO_2_ or CaO and bulk C12A7:e^−^ (formation enthalpy: 318 kJ mol^−1^; refs 41, 42, 43). In addition, extra-framework H^−^ ions can be formed by the reaction of C12A7:O^2−^ or C12A7:e^−^ with hydrogen gas. The formation enthalpies of C12A7:H^−^ in these processes are −367 and −434 kJ mol^−1^, respectively^43^. The formation enthalpy from C12A7:e^−^ is larger by 67 kJ mol^−1^ than that from C12A7:O^2−^. To clarify behaviour of hydrogen in Ru/C12A7:e^−^, TPA and TPD of H_2_ were examined for Ru-deposited catalysts, and the results are shown in Fig. 4. Ru/CA catalyst cannot absorb H_2_ at all in a flow of H_2_–Ar gas. On the other hand, TPA results for Ru/C12A7:O^2−^ and Ru/C12A7:e^−^ show broad peaks at 623–873 K, and the latter has a larger peak than the former. Neutral hydrogen species, such as H^0^ and H_2_, are metastable in C12A7 because the cage wall is positively charged, while H^−^ ions are thermodynamically stable, as shown in Fig. 5. The amounts of H^−^ ions incorporated in Ru/C12A7:e^−^ and Ru/C12A7:O^2−^ were estimated to be 164.8 and 39.0 μmol g^−1^, respectively. Ru/C12A7:e^−^ is thermodynamically more favourable than Ru/C12A7:O^2−^ for hydrogen incorporation. It was confirmed that the hydrogen incorporation of Ru/C12A7:e^−^ begins at lower temperatures than that of C12A7:e^−^ without Ru (Supplementary Fig. 7), indicating that dissociative adsorption of H_2_ and spillover of H adatoms from Ru surface facilitate hydrogen incorporation. Figure 4b shows H_2_ TPD for Ru catalysts after the ammonia synthesis for 5 h at 633 K. Although H_2_ desorption is not observed from Ru/C12A7:O^2−^ and Ru/CA, Ru/C12A7:e^−^ shows H_2_ desorption peaks ~573–773 K. The amount of H^−^ anions in Ru/C12A7:e^−^ during actual reaction (N_2_–H_2_) at 633 K was estimated from H_2_ TPD data shown in Fig. 4c. The amount of incorporated hydrogen increases with reaction time at the initial stage of the reaction (0–5 h), reaching a plateau after 20 h. The H^−^ amount incorporated for 20 h was 8.6 μmol g^−1^, which is only 1% of the theoretical maximum (1,427.7 μmol g^−1^) corresponding to C12A7:H^−^. The results for Ru/C12A7:e^−^ exposed to H_2_–Ar flow at the same temperature are also displayed in Fig. 5c. In this case, hydrogen is accumulated in proportion to reaction time, and the H^−^ amount incorporated for 40 h reaches 730 μmol g^−1^, corresponding to 50% of the theoretical maximum. These results clearly demonstrate that fast ammonia formation on Ru surface limits hydrogen incorporation into C12A7:e^−^, and thereby keeps a high density of the cage electrons, preventing decrease in activity.

## Discussion

**Figure 6:**
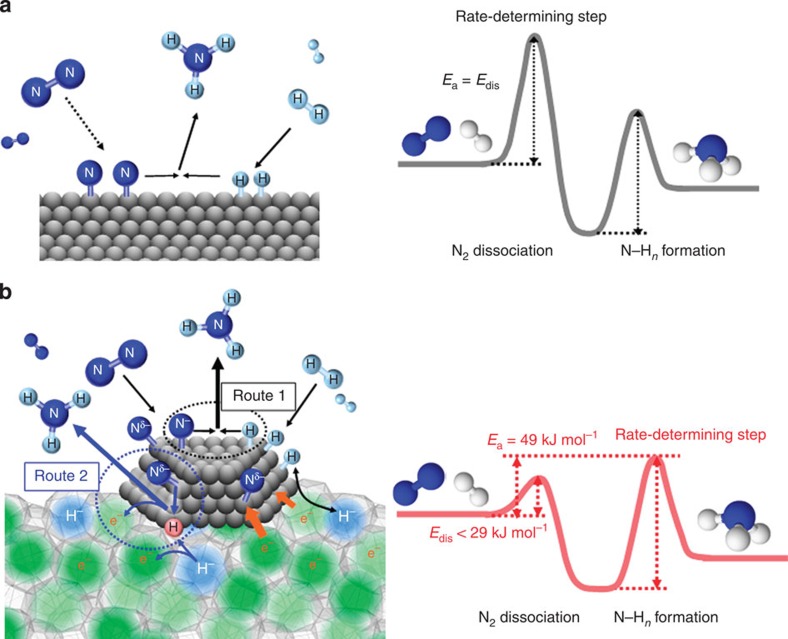
Proposed reaction mechanism and energy profile for ammonia synthesis. Reaction mechanism and energy profile for ammonia synthesis over (**a**) conventional catalyst and (**b**) Ru/C12A7:e^−^. (**a**) N_2_ and H_2_ react on the catalyst surface through a Langmuir–Hinshelwood mechanism to form NH_3_ in which N_2_ dissociation is the RDS. The energy barrier (*E*_dis_) for this step corresponds to the apparent activation energy (*E*_a_) for ammonia synthesis. As for Ru/C12A7:e^−^ (**b**), the rate-limiting step is not N_2_ dissociation but the formation of N–H_*n*_ species. NH_3_ is formed through the Langmuir–Hinshelwood mechanism (route 1) and the direct reaction of N adatoms with H radicals (nascent hydrogen) derived from cage H^−^ anions (route 2). *E*_a_ is determined by the difference between the top of the barrier for N–H_*n*_ formation and the energy level of reactant molecules (N_2_ and H_2_).

A reaction mechanism for ammonia synthesis over Ru/C12A7:e^−^, proposed on the basis of the above results, is illustrated in Fig. 6. For all conventional catalysts, N_2_ dissociation barrier (*E*_dis_) is the highest among all elementary steps in ammonia synthesis, resulting that the dissociative N_2_ adsorption is the RDS. In contrast, the reaction on Ru/C12A7:e^−^ is not limited by this step. This dissociation of the N≡N bond enhances the formation of N adatoms on the surface, resulting in the value 0.5 of the reaction order for N_2_, as expected for the N_2_ exchange reaction. Instead, the rate-limiting step of the electride catalyst is in the formation of N–H_*n*_ species. Dissociative H_2_ adsorption proceeds in parallel to N_2_ cleavage, increasing concentration of H adatoms on the Ru surface. A part of H adatoms moves into C12A7:e^−^ to provide a balance between H^−^ storage reaction (H^0^+e^−^→H^−^) and H atom release reaction (H^−^→H^0^+e^−^). H^−^ anions are not accumulated in proportion to reaction time unlike the case of Ru-free C12A7:e^−^ and the concentration is kept at ca. 1% of the theoretical maximum, due to fast ammonia formation derived from N adatoms with a high density and H atom release reaction. Such a dynamic mechanism makes it possible to keep high cage electron density and high catalytic performance. In addition, since both the initial agents and the reaction product are neutral, electrons transferred to nitrogen species through Ru then come back to the Ru/C12A7:e^−^ catalyst after the ammonia formation step completed and remain available for the next synthesis step^14^.

There are two possible routes to the formation of N–H_*n*_ species. The first route is a classical Langmuir–Hinshelwood mechanism between N and H adatoms on the Ru surface (route 1). The efficient reaction of N adatoms with a high density with H adatoms on the Ru surface consumes H adatoms, and the fast reversible hydrogen storage–release reaction balances the cage H^−^ with H adatoms on the Ru. Another possible route (route 2) is the direct reaction of N adatoms with H radicals, ‘nascent hydrogen’, from the cage H^−^ anions. Potential energy profile in ammonia synthesis for conventional catalysts, including commercial promoted iron catalysts, indicates that the formation of surface N–H_*n*_ species also requires a large activation energy because hydrogen has to react with N adatoms against strong N-transition metal interaction^3^,^30^. Ru has weaker interaction with N adatoms than Fe, so that conventional Ru-based catalysts exhibit higher catalytic performance around an atmospheric pressure compared with Fe-based catalysts^44^. Hydrogen adatoms have also to react with N adatoms to form N–H bonds, resisting strong interaction between H adatoms and Ru surfaces. In such a case, direct reaction of N adatoms with nascent hydrogen from the cage H^−^ would be energetically of greater advantage than the reaction among N and H adatoms on Ru surface. To further understand the processes involving H surface species, pathways leading to the formation of H^+^ on Ru/C12A7:e^−^ were also examined. These pathways include (1) H_2_→2H^+^+2e^−^ and (2) H_2_→H^+^+H^−^ reactions (Supplementary Fig. 8a,b). In the case of the former, H_2_ is homolytically dissociated to produce two H^+^ ions and two electrons. As such, these H^+^ ions may form O–H bonds with framework O^2–^ ions and electrons are introduced into the empty cages. In this case, H^−^ ions are not formed, which is contrary to the H_2_ TPD result (Fig. 4b). Therefore, the former possibility is ruled out. In the case of heterolytic H_2_ dissociation, H^+^ and H^−^ ions are formed on the catalyst surface. This phenomenon was found to take place on the surfaces of basic oxides, such as MgO, CaO and SrO^45^ and in stoichiometric C12A7 (ref. 46). H^−^ ion can be dissociated into two electrons and a proton, which is further converted to an OH^−^ ion via reaction with extra-framework oxide ion (O^2−^) as reported previously^47^. While there are no extra-framework O^2−^ ions in the bulk of Ru/C12A7:e^−^ catalyst, we cannot rule out the fact that the proton may be converted to an OH^–^ species via reaction with the framework oxygen at the surface of C12A7:e^−^ (Supplementary Fig. 8b). Hence, we cannot rule out the formation of transient H^+^ species through the heterolytic H_2_ dissociation. In any case, the formation of transient H^−^ ion is the important step of ammonia synthesis over Ru/C12A7:e^−^.

In summary, strong electron donation capability of C12A7:e^−^ allows ammonia formation along a new, highly efficient route where the activation energy for N_2_ cleavage is smaller than those of the subsequent N–H_*n*_ formation steps. Characteristics of fast reversible storage–release of hydrogen atoms on the surface of C12A7:e^−^ near Ru nanoparticles not only prevent hydrogen poisoning but also keep the cage electron density high near the surface, resulting in stable and highly active catalyst even under high pressure.

## Methods

### Catalyst preparation

C12A7:e^−^ powder samples were prepared by solid-phase reaction according to the following procedure. First, a mixture (Ca:Al=11:14) of CaCO_3_ and α-Al_2_O_3_ was ball-milled using a zirconia pot and yttria-stabilized zirconia balls (3 mm diameter) at a speed of 150 r.p.m. for 30 min. Then, this mixture was heated at 1573 K for 10 h in air, which led to the formation of intermixed C12A7 and CaO·Al_2_O_3_ (CA) powders, and then treated in a vacuum at 1273 K for 15 h. The resulting powder was mixed with Ca metal shot in a glove box filled with Ar gas, sealed in an evacuated silica tube and kept at 973 K for 15 h. The following reaction proceeds during this heat treatment: 0.8Ca_12_Al_14_O_33_+1.4CaAl_2_O_4_+Ca→Ca_12_Al_14_O_32_. Some of the Ca metal precipitates at the inner wall of the silica tube in this process. To compensate for this effect, we used twice the amount of Ca metal needed for this reaction. The glass tube was opened in the glove box and the reacted material was grinded with an agate mortar. Finally, the obtained powder was sealed in an evacuated silica tube and kept at 1,373 K for 2 h. C12A7:H^−^ was prepared by heating C12A7:e^−^ in a mixture of H_2_ and N_2_ gas flow (N_2_:H_2_=1:1) at 873 K for 12 h. CaO·Al_2_O_3_ (CA) was prepared by a reaction of CaCO_3_ and α-Al_2_O_3_ with a molar ration of 1:1 at 1,573 K for 20 h in an ambient air. The obtained powder was heated at 1,273 K for 15 h in a dynamically evacuated silica tube (~1 × 10^−4^ Pa) to eliminate water and hydroxyl groups on the surface. Ru-loaded samples were prepared by the following procedure. The sample powder and Ru_3_(CO)_12_ were sealed in an evacuated silica tube and were heated under the following temperature programme (2 K min^−1^ up to 313 K, hold for 1 h; in 2 h up to 343 K, hold for 1 h; in 2 h up to 393 K, hold for 1 h; and in 2.5 h up to 523 K, hold for 2 h; cooling down to ambient temperature). Since all Ru catalysts are deposited on the support by chemical vapour deposition method using Ru_3_(CO)_12_ as a precursor, zero-valence state of Ru is confirmed by X-ray photoelectron spectroscopy (XPS) analysis. The obtained sample was reduced *in situ* in a fixed bed flow system at 0.1 MPa in a stream of synthesis gas while the temperature was increased to 673 K at 1 K min^−1^.

### Catalytic reaction

N_2_ isotopic exchange study was conducted using a U-shaped glass reactor connected with a closed gas circulation system as reported elsewhere^10^,^28^. The mixture of ^15^N_2_ and ^14^N_2_ gases (total pressure: 20.0 kPa, ^15^N_2_: ^14^N_2_=1:4) was adsorbed on the catalyst without circulation at the reaction temperature until an adsorption equilibrium was achieved. The change in the composition of circulating gas was monitored by a quadrupole mass spectrometer (Bell Mass, BEL, Japan). The circulating pump placed in the system removes diffusional and adsorption/desorption limitations. The masses 28, 29 and 30 *m/z* were monitored as a function of time to follow the exchange. Ammonia synthesis was carried out in a fixed bed flow system with a synthesis gas (H_2_/N_2_=3) flow rate of 60 ml min^−1^. Limitations by diffusion and adsorption/desorption were avoided by using 0.2 or 0.025 g of catalyst (bed height<10 mm), which is similar to the conditions reported previously^37^. The reaction temperature was varied from 523 to 673 K, and the pressure was kept at 0.1 MPa. In addition, ammonia synthesis was performed using D_2_ instead of H_2_ to investigate the isotope effect. All kinetic experiments were carried out under far from equilibrium conditions (for example, the conversion level is smaller than 30% of that at equilibrium). The reaction orders with respect to N_2_ and H_2_ were obtained at a constant flow rate (60 ml min^−1^) using Ar gas as a diluents, and that for NH_3_ was determined with (3H_2_+N_2_) by changing the synthesis gas flow rate^14^. The produced ammonia was trapped by in a 5-mM sulfuric acid solution, and the amount of NH^4+^ generated in the solution was determined by ion chromatography (LC-2000 plus, JASCO).

### Measurement of the electron density of Ru/C12A7:e^−^

An iodometric titration method was used to confirm the presence of electrons and quantify the electron concentration (*N*_e_) in the Ru/C12A7:e^−^ catalyst. Approximately 10 mg of catalyst was dispersed in an aqueous I_2_ solution (5.0 × 10^−3^ M, 3 ml), and then 0.1 ml of HCl was poured into the solution. After confirming complete dissolution of the sample, the amount of residual I_2_ was titrated using sodium thiosulfate solution (5.0 × 10^−3^ M). Observation of the endpoint was enhanced by adding a few drops of starch solution, which induces a violet coloration. The average electron density was obtained from three independent measurements.

### Temperature-programmed absorption

TPA of H_2_ was analysed using a BELCAT-A instrument (BEL, Japan). Before measurements, the samples (~100 mg) were heated in an Ar stream (50 ml min^−1^) at 393 K for 90 min to remove water adsorbed on the surface, followed by cooling in a stream of Ar. Then, the sample was heated (2 K min^−1^) in a stream of 4.8% H_2_/Ar mixture, and the consumption of H_2_ was monitored by a thermal conductivity detector (TCD) and mass spectrometer (Bell Mass, BEL, Japan).

### Temperature-programmed desorption

TPD of H_2_ was performed using the same instrument as TPA experiment. Before the measurements, the sample was heated under a mixture of H_2_ and N_2_ (H_2_/N_2_=3, flow rate: 60 ml min^−1^, pressure, 0.1 MPa; temperature, 633 K, time, 5 h), which is the same reaction condition as that of ammonia synthesis. After cooling to room temperature, the sample was exposed to air to remove hydrogen adatoms on the Ru surface. Then, the sample was heated in an Ar stream (50 ml min^−1^) at 393 K for 90 min to remove water adsorbed on the surface and was heated (10 K min^−1^) in an Ar stream (50 ml min^−1^), and the concentration of H_2_ was monitored by a thermal conductivity detector (TCD) and mass spectrometer (Bell Mass, BEL, Japan).

### Computational modelling

*Ab initio* simulations were carried out using the density functional theory (DFT), with the generalized gradient approximation functional of Perdew–Burke–Ernzerhof^48^, and the projected augmented waves method^49^ implemented in the Vienna *ab initio* simulation package^50^,^51^. The plane-wave basis set cutoff was set to 500 eV. The C12A7 surface was modelled using a quasi-two-dimensional slab^52^. The super-cell parameters in the *x*–*y* plane and in the direction perpendicular to the surface were fixed at 12 and 24 Å, respectively. As the near-surface region is partially disordered^50^, the calculations were carried out for the G point of the Brillouin zone only. Atomic charges were determined using the Bader analysis of the charge density distribution^53^.

## Author contributions

H.H. proposed an idea of this subject, and M.H. and H.H. directed the entire project. M.K., S.K. and Y.I. performed the synthesis, characterization and catalytic testing of Ru/C12A7:e^−^. P.V.S. and N.K. carried out *ab initio* calculations. All authors discussed the results and commented on the study. M.K, M.H., P.V.S. and H.H. co-wrote the manuscript.

## Additional information

**How to cite this article**: Kitano, M. *et al*. Electride support boosts nitrogen dissociation over ruthenium catalyst and shifts the bottleneck in ammonia synthesis. *Nat. Commun.* 6:6731 doi: 10.1038/ncomms7731 (2015).

## Supplementary Material

Supplementary Information Supplementary Figures 1-8 and Supplementary Table 1

## Figures and Tables

**Table 1:** Activities of N_2_ isotopic exchange reaction.

**Catalyst**	**Surface area (m**^**2**^** g**^−**1**^**)**	Ru loading (wt%)*	**Dispersion (%)†**	**Particle size (nm)**†	**Catalytic activity**		**TOF (s**^−**1**^**)**‡	***E***_**a**_ **(kJ mol**^−**1**^**)**§
					**mmol g**^−**1**^** h**^−**1**^||	**mmol m**^−**2**^** h**^−**1**^¶		
Ru/C12A7:e^−^	1.0	1.2	3.2	41.3	2.73	2.73	0.200	58
Ru/C12A7:O^2−^	1.0	1.2	3.4	39.2	0.42	0.42	0.029	133
Ru-Cs/MgO	12.0	6.0	18.6	7.2	1.33	0.11	0.003	139
Ru/CA	1.2	1.2	3.2	41.3	0.07	0.06	0.005	154

Ru, ruthenium; TOF, turnover frequency.

Reaction conditions: catalyst (0.5 g), reaction gas (^15^N_2_: ^14^N_2_=1: 4), pressure (26.7 kPa) and reaction temperature (633 K).

^*^Ru content was determined by ICP-AES.

^†^Dispersion and particle size were calculated on the basis of CO chemisorption values, assuming spherical metal particles and the stoichiometry of Ru/CO=1.

^‡^TOF was calculated from the reaction rate divided by the number of CO atoms chemisorbed on the Ru surfaces.

^§^*E*_a_ is the apparent activation energy calculated from Arrhenius plots of the reaction rate in the temperature range of 613–673 K.

^||^Catalytic activity was described as reaction rate per catalyst weight.

^¶^Catalytic activity was described as reaction rate per surface area.

**Table 2:** Activation energy of NH_3_ synthesis and N_2_ desorption.

**Catalyst**	**Activation energy (kJ mol**^−**1**^**)**			**Δ**E*
	**NH**_**3**_ **synthesis**	**N**_**2**_**exchange (N**_**2**_**desorption)**	**N**_**2**_ **TPD (N**_**2**_**desorption)**	
Ru/C12A7:e^−^	49	58	64†	9
Ru/C12A7:O^2−^	104	133		29
Ru-Cs/MgO	99	139	137‡	40
Ru/CA	118	154		36
Ru/MgO	80		158‡	78

TPD, temperature-programmed desorption.

^*^Δ*E* is difference in the activation energy between N_2_ exchange reaction and ammonia synthesis.

^†^From ref. 31.

^‡^From ref. 27.

**Table 3:** Orders of reaction for ammonia synthesis over various Ru catalysts.

**Catalyst**	**N**_**2**_ **order (*****α*****)**	**H**_**2**_ **order (*****β*****)**	**NH**_**3**_ **order (*****γ*****)**
Ru/C12A7:e^−^	0.46	0.97	−1.00
Ru/C12A7:O^2−^	1.00	0	−0.25
Ru/C12A7:H^−^	1.00	−0.63	−0.60
Ru/CaO·Al_2_O_3_	0.82	−0.38	−0.68
Ru/MgO*	0.82	−0.38	−0.68
Ru-Cs/MgO†	1.0	−0.43	−0.12
Ru powder†	0.96	−0.72	−0.15
Co_3_Mo_3_N‡	0.99	0.8	−1.34
Co-Ba/C§	0.9	1.2	−0.9
Fe(KM1)§	0.9	2.2	−1.5

^*^From ref. 14.

^†^From ref. 34.

^‡^From ref. 35.

^§^From ref. 36.
