# Supplementary material for: Electride support boosts nitrogen dissociation over ruthenium catalyst and shifts the bottleneck in ammonia synthesis
Source: Nat Commun. 2015 Mar 30;6:6731. doi: 10.1038/ncomms7731 (PMC4389256; doi:10.1038/ncomms7731)
Supplement: Supplementary Information — Supplementary Figures 1-8 and Supplementary Table 1 [file ncomms7731-s1.pdf]

SUPPLEMENTARY INFORMATION

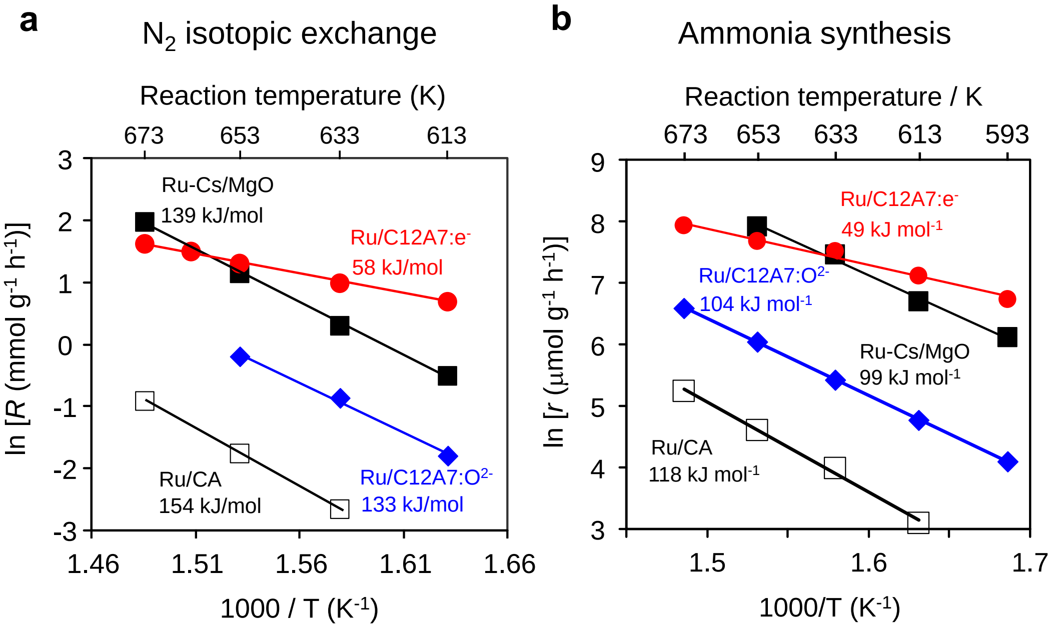

Supplementary Figure 1 (**a**) Temperature dependence of the rate of N_2 isotopic exchange reaction over various Ru catalysts at 26.7 kPa ($^{15}N_2$: $^{14}N_2$ = 1 : 4). (**b**) Temperature dependence of the rate of ammonia synthesis over various 1 wt% Ru-loaded catalysts at an atmospheric pressure (catalyst = 0.2 g, H_2:N_2 = 3:1, flow rate = 60 mL min^{-1}).

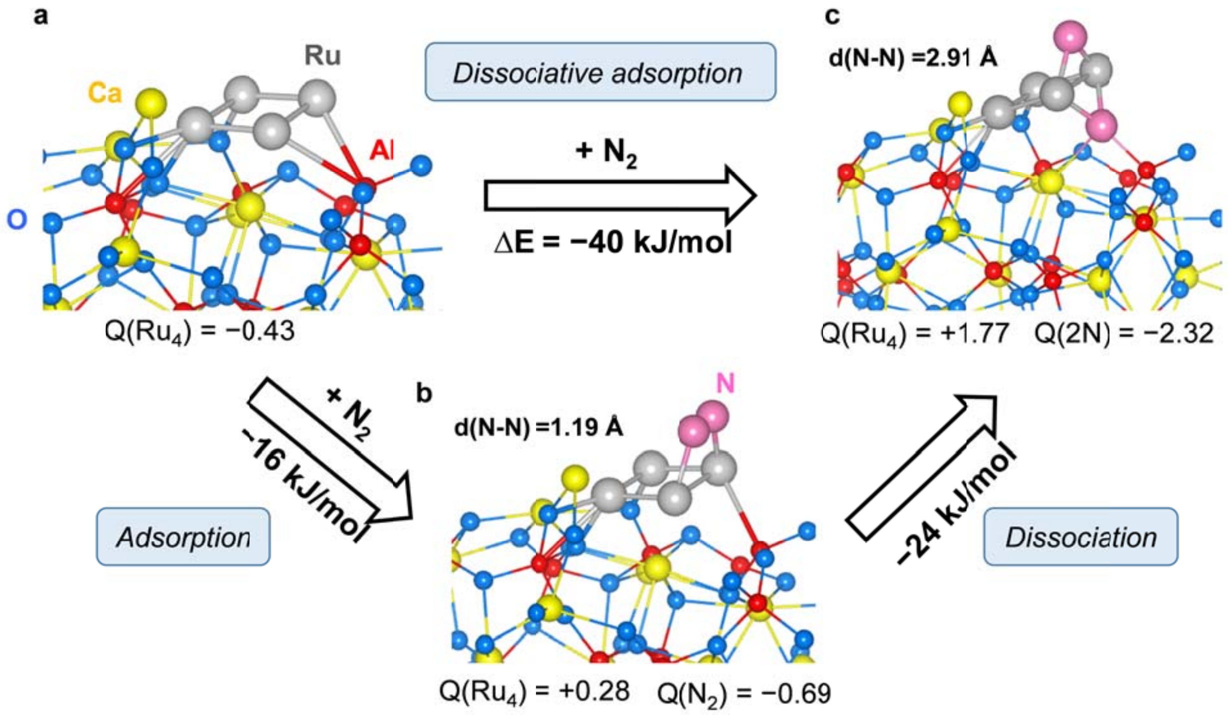

Supplementary Figure 2 The most stable geometrical configurations of Ru_4 cluster and Ru_4N_2 complex on C12A7:O^{2-} surfaces obtained using *ab initio* simulations. (A) The negative charge of the Ru_4 cluster supported on C12A7:O^{2-} is associated with the formation of Ru-O bonds. (B) N_2 molecule is adsorbed on Ru sites with the adsorption energy of 16 kJ mol^{-1}, and the resulting N-N bond length, d(N-N), is 1.19 Å. (C) N_2 dissociation proceeds with the energy gain of 24 kJ mol^{-1}, and the resulting N-N distance is 2.91 Å. ΔE corresponds to dissociative adsorption, i.e., the sum of the energy of the N_2 adsorption on C12A7-supported Ru and the energy gain due to N_2 dissociation. Q(R_4), Q(N_2), and Q(2N) represent Bader charge on Ru_4 cluster, N_2 molecule, and resulting two N atoms, respectively.

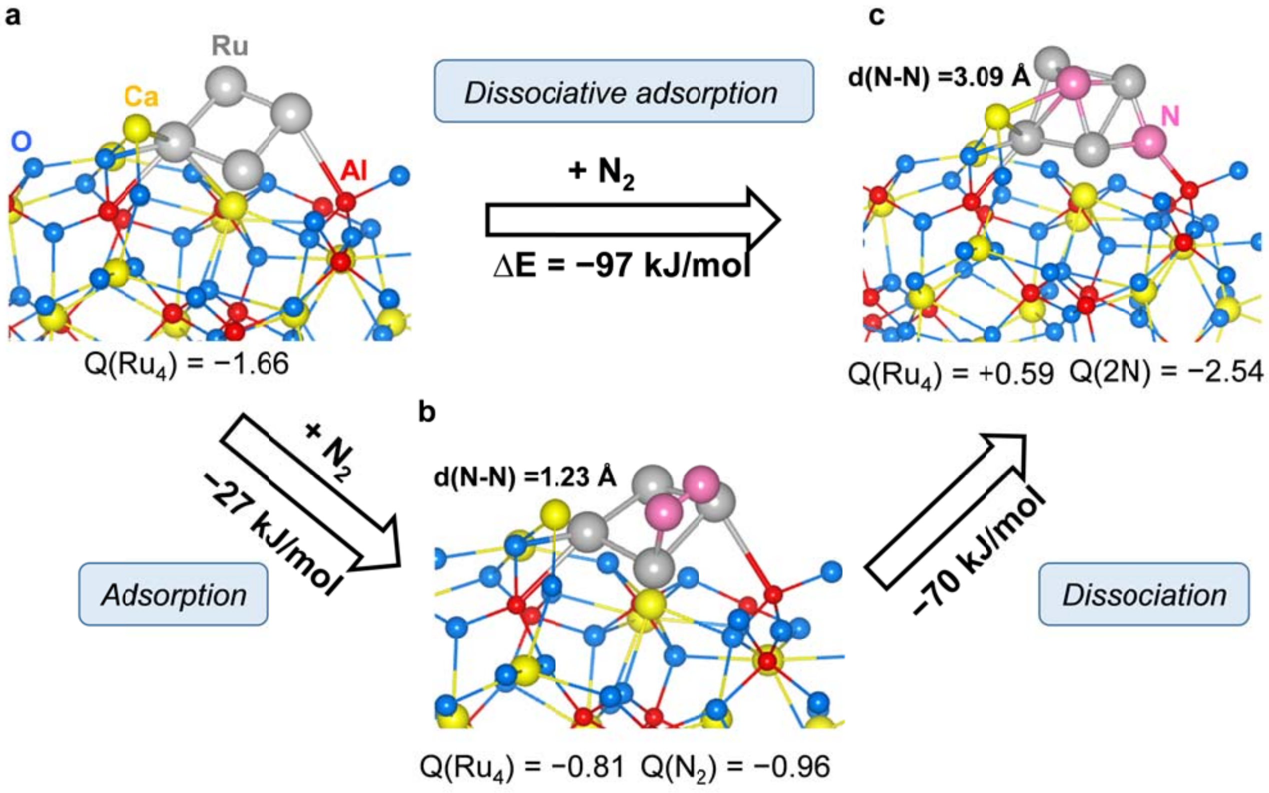

Supplementary Figure 3 The most stable geometrical configurations of Ru_4 cluster and Ru_4N_2 complex on C12A7:e^- surfaces obtained using *ab initio* simulations. (A) The negative charge of the Ru_4 cluster supported on C12A7:e^- is due to both the charge transfer from the substrate to Ru_4 and the charge density distribution associated with the formation of Ru-O bonds. (B) N_2 molecule is adsorbed on Ru sites with adsorption energy of 27 kJ mol^{-1}, and the resulting N-N bond length, d(N-N), is 1.23 Å. (C) N_2 dissociation proceeds with the energy gain of 70 kJ mol^{-1}, and the resulting N-N distance is 3.09 Å. ΔE corresponds to dissociative adsorption, i.e., the sum of the energy of the N_2 adsorption on C12A7-supported Ru and the energy gain due to N_2 dissociation.

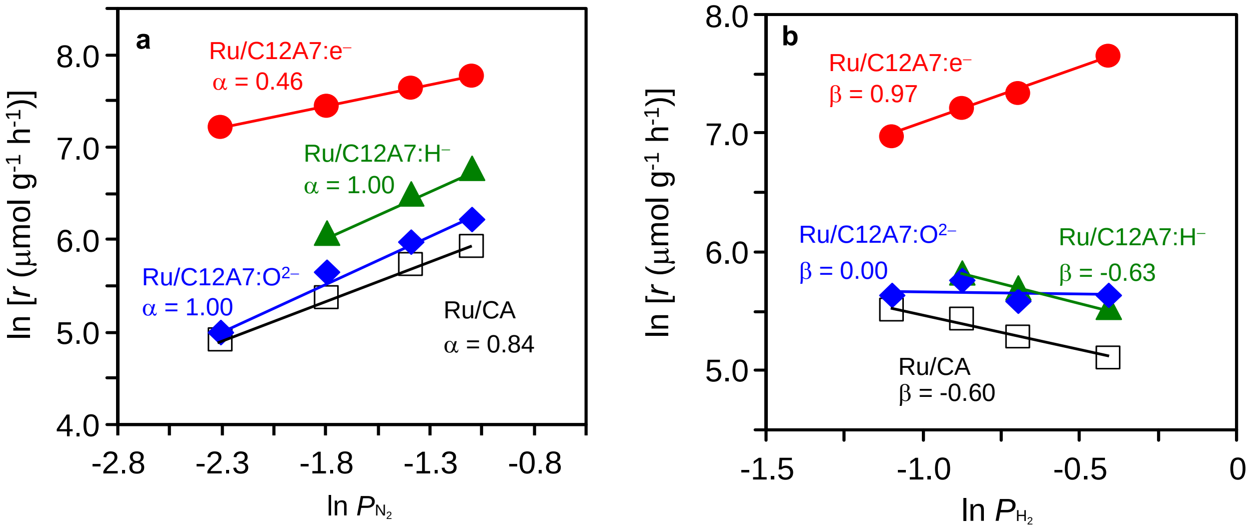

Supplementary Figure 4 Dependence of NH_3 synthesis rate on the partial pressures of (a) N_2 and (b) H_2 under atmospheric pressure. α and β represent the reaction orders for N_2 and H_2 in Eq. 2, respectively.

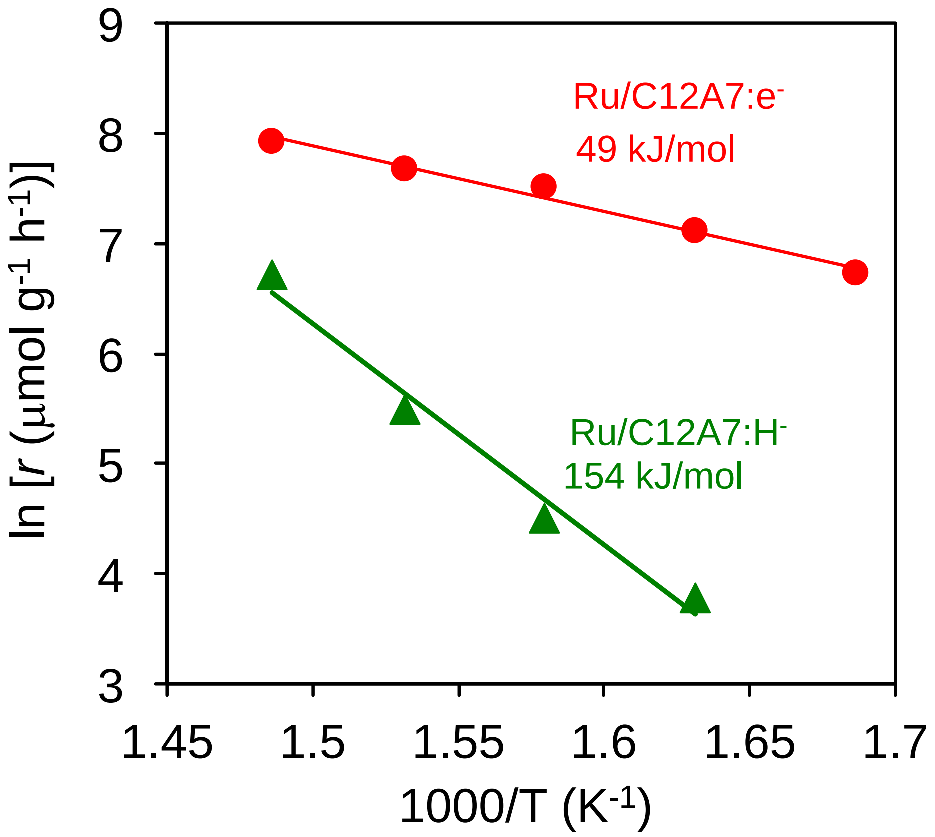

Supplementary Figure 5 Temperature dependence of the rate of ammonia synthesis over various 1 wt% Ru-loaded catalysts at an atmospheric pressure (catalyst = 0.2 g, H_2:N_2 = 3:1, flow rate = 60 mL min^{-1}).

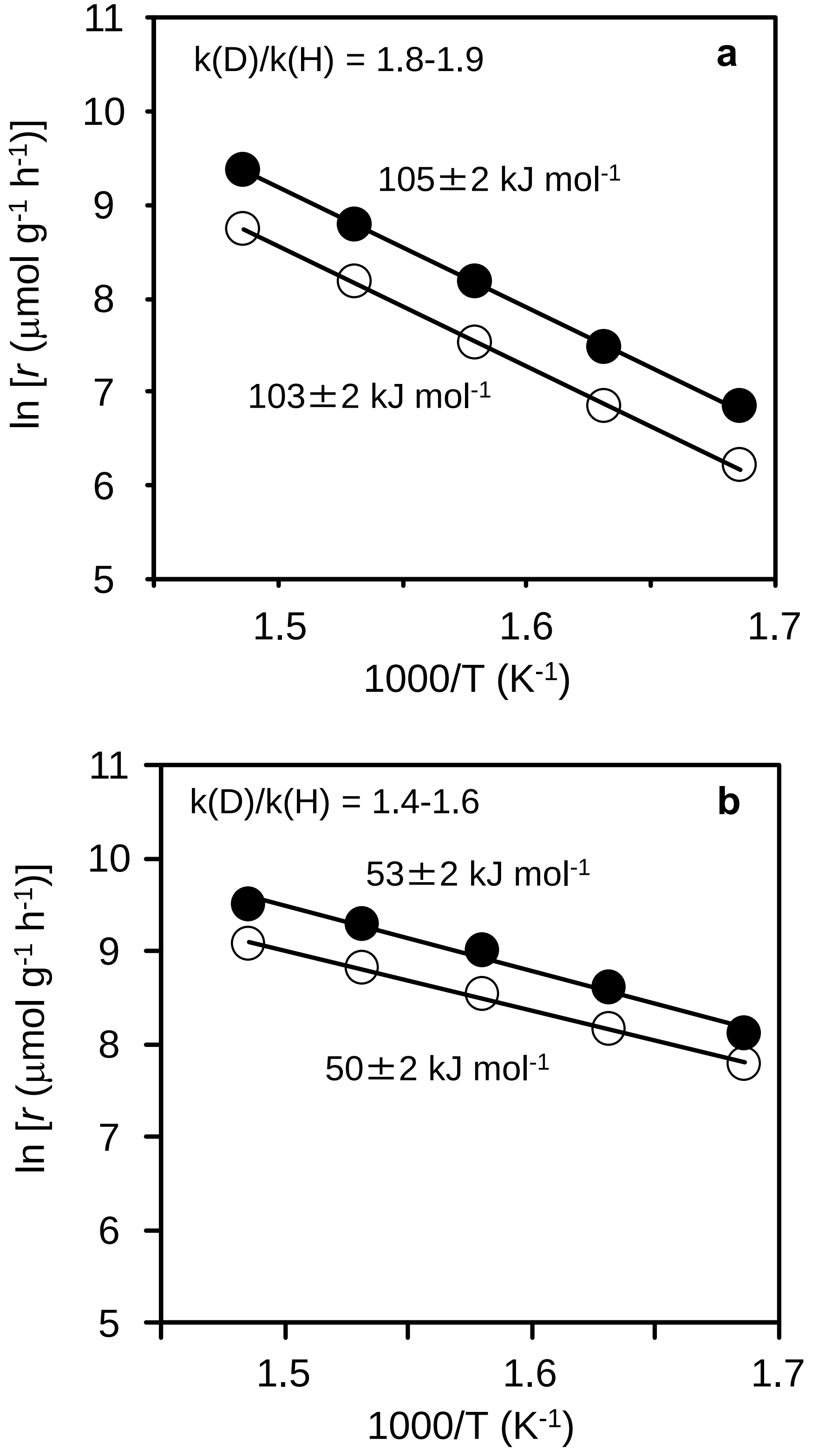

Supplementary Figure 6 Comparison of rates of ammonia synthesis with H_2 (open circles) and D_2 (filled circles) over (a) 1wt% Ru-Cs/MgO and (b) 1wt% Ru/C12A7:e^-. (catalyst = 0.025 g, H_2(or D_2):N_2 = 3:1, flow rate = 60 mL min^{-1}).

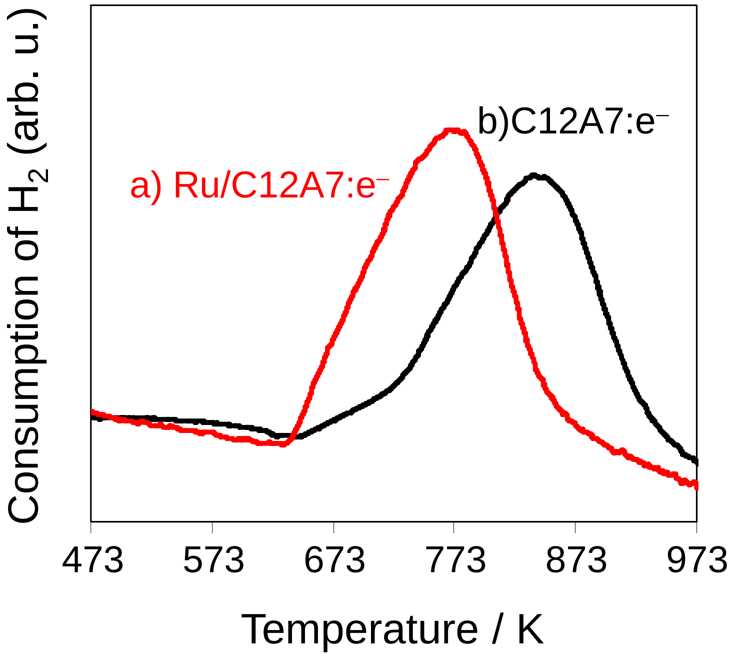

Supplementary Figure 7 H_2 TPA profiles of a) Ru/C12A7:e^{-} and b) C12A7:e^{-}. The TPA experiment was performed with a dilute mixture of H_2 (5%) in Ar using a total flow of 10 ml min^{-1} and a heating rate of 2 K min^{-1}.

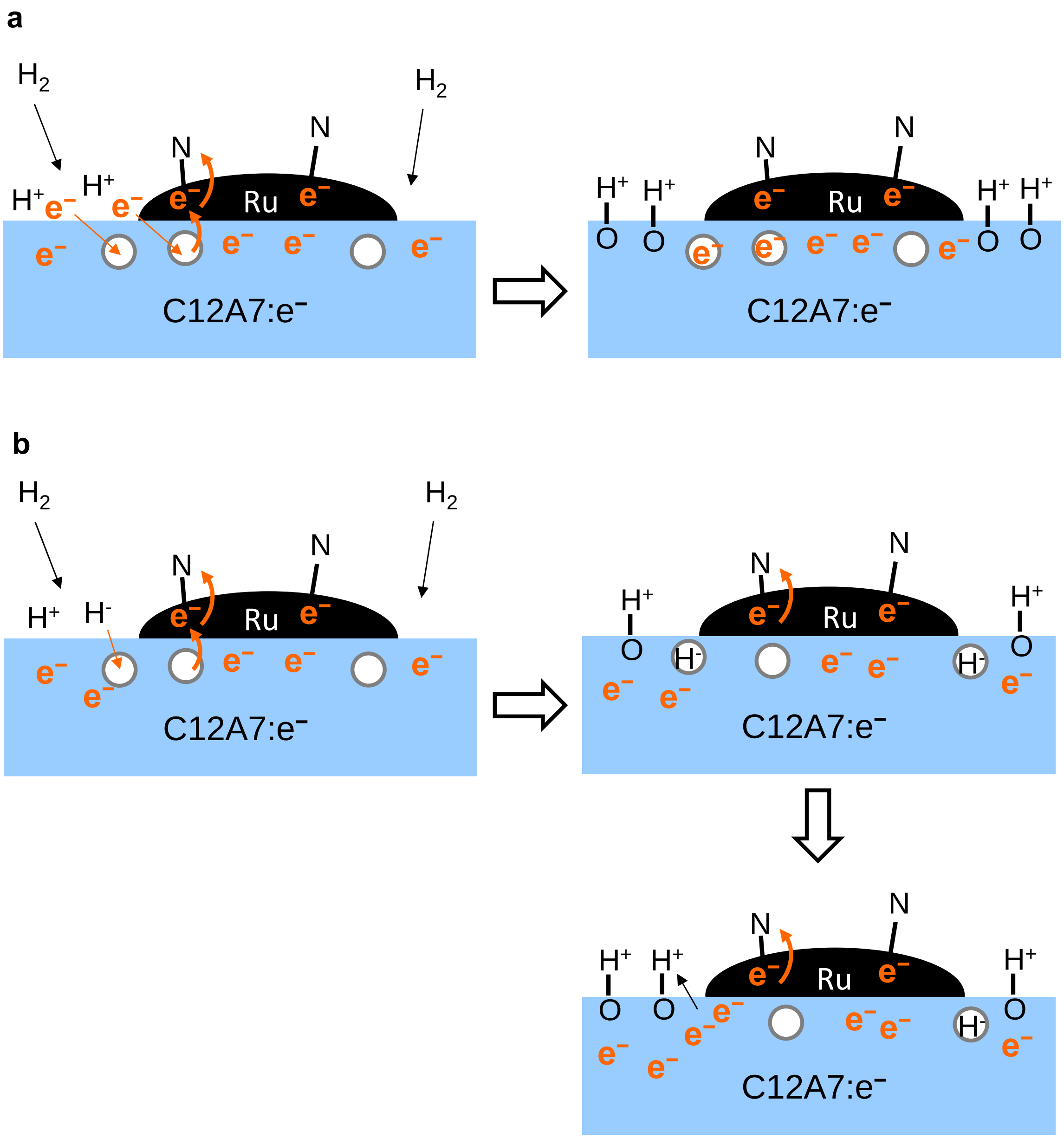

Supplementary Figure 8 (**a**) Reaction pathway 1: H_2 is dissociated on Ru/C12A7:e^- to produce two OH (formally H^+ state) species (homolytic dissociation). (**b**) Reaction pathway 2: H_2 is dissociated on Ru/C12A7:e^- to produce OH (formally H^+ state) and H^- ions (heterolytic dissociation).

Supplementary Table 1 Catalytic performance of various Ru catalyst for ammonia synthesis reaction at 633 K

Catalyst	Surface area ($m^2\ g^{-1}$)	Catalytic activity		Ea ($kJ\ mol^{-1}$)[f]
		($mmol\ g^{-1}\ h^{-1}$)[a]	($mmol\ m^{-2}\ h^{-1}$)[b]	
Ru/C12A7:e^-	1.0	1.82	1.82	49
Ru/C12A7:O^{2-}	1.0	0.25	0.25	104
Ru-Cs/MgO	12	1.72	0.14	99
Ru/CA	1.2	0.15	0.13	118

[a,b]Reaction conditions: catalyst = 0.2 g, H_2:N_2 = 3:1, flow rate = 60 mL min^{-1}, pressure = 0.1 MPa. Catalytic activity was described as ammonia synthesis rate per (a) catalyst weight or per (b) surface area.
